# Supplementary material for: What combination of interventions can optimise HIV prevention for adolescent girls and young women? Cohort analysis of DREAMS participation in urban and rural Kenya
Source: PLOS Glob Public Health. 2025 Oct 7;5(10):e0005272. doi: 10.1371/journal.pgph.0005272 (PMC12503341; doi:10.1371/journal.pgph.0005272)
Supplement: S1 Text — (DOCX) [file pgph.0005272.s001.docx]

**DAGS Nairobi**

**Knowledge of HIV status**


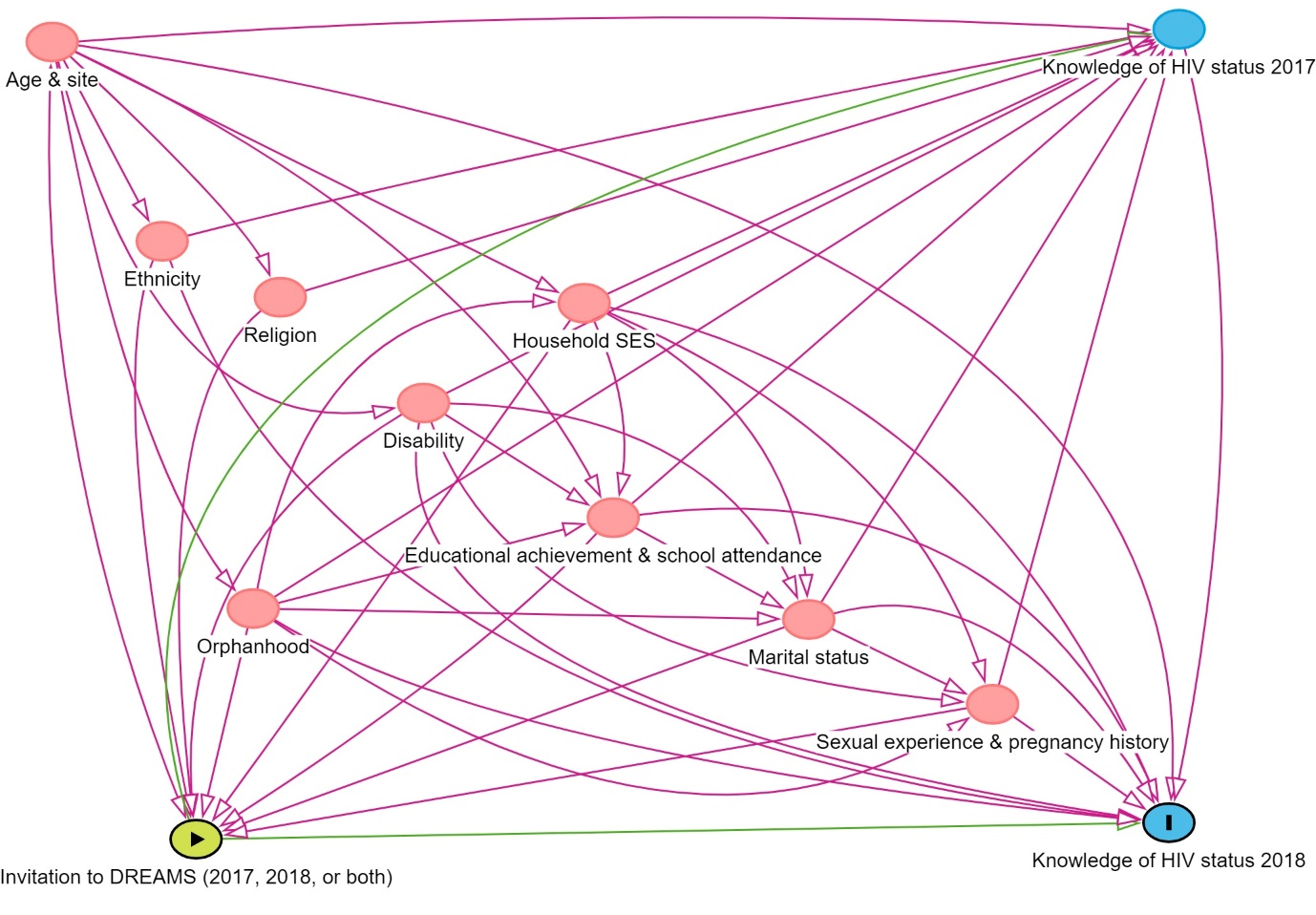


**Condomless sex**


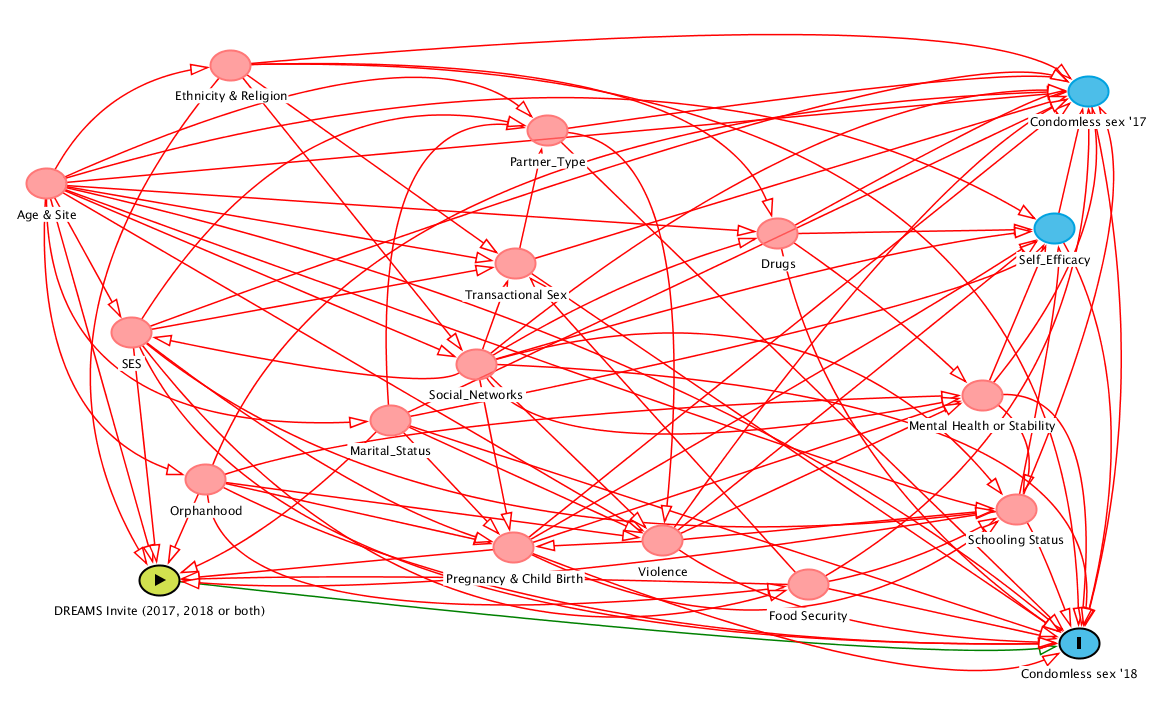


**Number of partners**


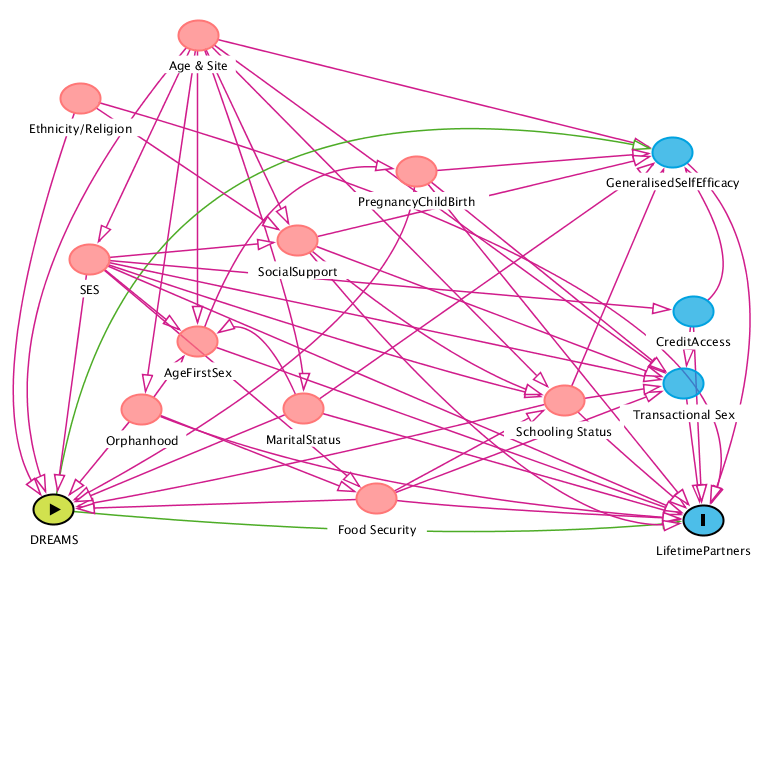


**DAGS Gem**

**Knowledge of HIV status**

**Condomless sex**

Social support
