# Supplementary material for: What combination of interventions can optimise HIV prevention for adolescent girls and young women? Cohort analysis of DREAMS participation in urban and rural Kenya
Source: PLOS Glob Public Health. 2025 Oct 7;5(10):e0005272. doi: 10.1371/journal.pgph.0005272 (PMC12503341; doi:10.1371/journal.pgph.0005272)
Supplement: S2 Text — (DOCX) [file pgph.0005272.s002.docx]

**Causal inference assumptions and interpretations**

To ensure the propensity score analyses produce valid causal estimates for the outcomes, five key assumptions are necessary:

1. **no interference (Stable Unit Treatment Value Assumption (SUTVA)**

This assumption implies that the outcome of one individual is not affected by the treatment status of another. In this study, we assume that an AGYW’s exposure to DREAMS does not influence the outcomes of another AGYW. This is a reasonable assumption, as the primary outcomes; knowledge of HIV status, condomless sex, social support, self-efficacy, and number of lifetime partners are largely shaped by individual experiences and broader contextual factors. Therefore, it is unlikely that one participant’s exposure to DREAMS would substantially affect another’s outcomes. However, in rare cases where two or more AGYW reside in the same household, there may be some spillover effects, such as shared knowledge or attitudes resulting from one participant receiving DREAMS interventions. Despite this possibility, such instances are limited within the study context, and we consider the assumption of no interference to be plausible

1. **Positivity assumption.**

For valid estimation, every participant must have a non-zero probability of receiving each level of treatment, given their covariates. In this study, we ensured positivity by checking the distribution of estimated propensity scores across exposure groups. The propensity scores distribution revealed substantial overlap. There were very minimal instances with no overlap. Selection bias is more common in observational studies and can rarely be perfectly representative due to factors such as refusals. However, the positivity assumption was met based on the propensity score distribution

1. **Consistency**

This assumption requires that exposure to DREAMS is clearly defined, such that any variations in receiving DREAMS would not result in a different outcome. In our study, DREAMS exposure was defined as receiving either a “complete” or “partial” package of core interventions. The exposure clearly distinguishes those who either received a “complete” or “partial” package of core interventions. We also ensured that the definition was consistent across study sites. DREAMS is a combined package intervention, and some modifications were allowed. Despite some context specific adjustments, implementation and delivery was conducted using the same strategies in each site within a similar time period. Thus, impact of these variations is likely to be minimal and thus consistency assumption is plausible.

1. **Conditional exchangeability**

This assumption states that given a set of covariates, the exposed and unexposed groups should be interchangeable. The validity of this assumption was achieved through the construction of a close to accurate Directed Acyclic Graphs(DAGs), which resulted in an adequate set of adjustment. Additionally, the availability and correct measurement of the variables identified as confounders equally validated this assumption. Elimination of unmeasured confounding entirely is unlikely, but the bias was minimised to a great extent by the adjustment of the minimal adjustment set.
