# Supplementary material for: What combination of interventions can optimise HIV prevention for adolescent girls and young women? Cohort analysis of DREAMS participation in urban and rural Kenya
Source: PLOS Glob Public Health. 2025 Oct 7;5(10):e0005272. doi: 10.1371/journal.pgph.0005272 (PMC12503341; doi:10.1371/journal.pgph.0005272)
Supplement: S3 Text — (DOCX) [file pgph.0005272.s003.docx]

**Study Procedures to safeguard confidentiality**

We implemented several procedures to ensure confidentiality and protect participants' privacy, particularly when addressing sensitive topics.

Trained and experienced interviewers: All participants were interviewed by trained data collectors who were fluent in the local language, familiar with the cultural context, and experienced in handling sensitive topics. Interviewers were trained on ethical research practices, including maintaining confidentiality and creating a non-judgmental and safe environment for AGYW. Before each interview, participants were informed about the purpose of the study, their right to decline or skip any question, and the strict confidentiality of their responses.

Anonymized data collection: No personally identifiable information (e.g., names, addresses, phone numbers) was collected in the main dataset used for analysis. Each participant was assigned a unique study ID, and all analyses were conducted using anonymized datasets.

Data were collected using secure electronic tablets with password protection and encrypted data storage. The system offered enhanced security compared to paper-based methods, as it reduces the risk of physical data loss or unauthorized access. Data were synchronized daily to a secure server with restricted access.

Access to the data was limited to authorized study personnel only. Data were stored on password-protected and encrypted servers, and all data handling procedures adhered to institutional and national data protection guidelines.

**Methods employed to verify response accuracy and specify any quality-control metrics applied**

To ensure the accuracy of AGYW responses, we implemented multiple quality assurance procedures during data collection. All data collectors underwent intensive training on the study protocol, questionnaire content, ethical conduct, and appropriate probing techniques. Mock interviews and field practice sessions were conducted during the training to ensure consistency and correct interpretation of questions.

We used a tablet-based electronic data capture system programmed with built-in skip patterns, logic checks, and range validations to prevent illogical or inconsistent responses. These automated checks minimized data entry errors at the point of collection.
Data collected each day were synchronized to a secure central server and study team conducted quality checks. These included checking for missing data, inconsistencies, and outlier values. Any flagged records were reviewed and, when needed, followed up with the interviewer. Field supervisors regularly conducted spot checks and random checks to confirm that interviews had been conducted as reported, and that protocols were being followed.
Regular debriefing meetings were held with field staff to discuss challenges, clarify questionnaire items, and share feedback from data quality reviews. Interviewers received refresher guidance or corrective instructions as needed.

**Adaptations made to account for developmental differences across early, middle, and late adolescence.**

Although the broader DREAMS programme targets AGYW aged 10–24 years, this analysis focused on a sub-sample of AGYW aged 13–22 years. Questions about sexual behavior, including lifetime number of sexual partners, were only asked if the AGYW indicated that they had ever had sex. This minimised unnecessary or inappropriate questioning among younger participants who had not initiated sexual activity. Sensitive items were phrased using appropriate and non-judgmental language. Interviewers received training on how to approach sensitive topics with adolescents of different ages, including strategies for building rapport, ensuring privacy, and adjusting tone and pace based on the age of the AGYW. Interviews were conducted in private settings to help participants feel comfortable and safe when responding to sensitive questions. By implementing these adaptations, we aimed to create an appropriate environment for AGYW while maintaining the integrity of data collection.

**Storage of consent forms**

Signed consent forms were securely stored in locked filing cabinets at the study site offices with access limited to authorized study personnel. These forms were not linked to the analytical dataset, which contained only anonymized data, to ensure participant confidentiality
