# Supplementary material for: What combination of interventions can optimise HIV prevention for adolescent girls and young women? Cohort analysis of DREAMS participation in urban and rural Kenya
Source: PLOS Glob Public Health. 2025 Oct 7;5(10):e0005272. doi: 10.1371/journal.pgph.0005272 (PMC12503341; doi:10.1371/journal.pgph.0005272)
Supplement: Attachment — Submitted filename: PLOS Reviewers Comments.docx [file pgph.0005272.s014.docx]

## **What combination of interventions can optimise HIV prevention for adolescent girls and young women? Cohort analysis of DREAMS participation in urban and rural Kenya.**

**Overall comments**

The authors provide a comprehensive evaluation of a combination of interventions that can optimize HIV prevention among adolescent girls and young women (AGYW) in Kenya. This work contributed to the evidence base on HIV programming in resource-limited settings. However, this manuscript could be made stronger and could benefit from streamlining of the content and improved clarity.

See comments below for consideration

**Introduction**

Clearly outlined with excellent contextual information. However, it’s a bit long and would benefit from a reduction in some content that can be moved to the methods section. For example, the DREAMS selection process and package lists could be moved to the methods section.

**Methods**

Well-structured design applied across two diverse settings. Detailed explanation and account of the methods provided. While the use of statistical techniques is appropriate and required, the analysis section is overly lengthy and somewhat technical, which may limit some audiences. Perhaps consider summarizing the key analytic steps more concisely to improve clarity and reader engagement.

Ethical considerations-where were the consent forms stored?

**Discussion**

The findings are appropriately synthesized and interpreted within the broader literature.

Line 517-555 provides a summary of the findings, though long. Please revise to be more concise and focus on the key findings.

Line 551-552- Why do you think the partial package was effective than the complete package in Nairobi?

Line 586-593 Briefly discuss how the intervention tailoring by age can influence these patterns

Line 594-596: Provide details on how the sustainability of the effects in the long term may rely on family, community, and institutional support. Integration?
