# Supplementary material for: What combination of interventions can optimise HIV prevention for adolescent girls and young women? Cohort analysis of DREAMS participation in urban and rural Kenya
Source: PLOS Glob Public Health. 2025 Oct 7;5(10):e0005272. doi: 10.1371/journal.pgph.0005272 (PMC12503341; doi:10.1371/journal.pgph.0005272)
Supplement: Attachment — Submitted filename: Response to reviewers.docx [file pgph.0005272.s016.docx]

Collins Otieno Asweto, PhD
Academic Editor
PLOS Global Public Health

Re: Manuscript ID PGPH-D-25-00897

What combination of interventions can optimise HIV prevention for adolescent girls and young women? Cohort analysis of DREAMS participation in urban and rural Kenya.

Thank you for sharing the peer reviews of our manuscript and inviting us to submit a revision. We are pleased that you and the reviewers saw the value of this work and appreciate their feedback.

We have responded to each comment below and uploaded revised versions (clean and with tracked changes) for your consideration.

We look forward to your response.

Sincerely,

Faith Magut, on behalf of the authors

Reviewer #1: Overall, this study is excellent and acceptable for publication in PLOS Global Public Health. Several minor comments should be considered to improve the clarity and strength of the article. Please ensure that grammatical errors are addressed throughout the manuscript.

Abstract
- Please describe the DREAMS intervention.

We have included a short description of DREAMS intervention in the abstract. In brief, DREAMS partnership (“Determined, Resilient, Empowered, AIDS-free, Mentored and Safe”) is a multi-component HIV prevention program combining behavioural, biomedical and structural interventions, launched in 2015 by PEPFAR with the aim of reducing HIV incidence among AGYW. (Line 26-27, Page 2)

- Should mention the type of statistical approaches used with the causal inference framework (i.e., descriptive statistics, logistic regression).

We have revised the manuscript to include more details on the statistical approaches used within the causal inference framework. Specifically, we used descriptive statistics to summarise the characteristics of the study population and used propensity score logistic regression adjustment to estimate the effect of the DREAMS interventions on the outcomes controlled for confounding. We have clarified in the abstract that we used propensity score-adjusted logistic regression to estimate the causal effect of DREAMS on outcomes under three counterfactual scenarios: all AGYW accessed the complete package, all accessed a partial package, or none were invited to DREAMS (Line 39-40, page 2)

- There should be a mention of the study design (i.e., a cohort study) in the first sentence of the methods.

Thank you for the comment. We have included the description of the study design. To clarify, we conducted a prospective cohort study among AGYW aged 13–22 years, randomly selected in rural Gem and urban Nairobi informal settlements and followed up from 2017/2018 to 2019. (Line 31, page 2)
- Could you clarify in the abstract that Gem is a rural area and Nairobi is an urban area? The writing in the abstract currently suggests that Nairobi is also a rural area.

Thank you for the suggestion. We have revised the abstract to clarify that Gem is a rural area and Nairobi informal settlements represent an urban setting. (Line 32, page 2)

- How were people recruited (i.e., online, in-person)? Purposeful sampling/Targeted sampling from settlements?

AGYW were randomly selected from a sampling frame drawn from the Health and Demographic Surveillance System (HDSS) in each study site. Data were collected in person through face-to-face interviews, using an electronic data collection tool administered by trained and experienced field interviewers who were conversant with the local context. Field staff were recruited based on their academic qualifications, prior experience in both quantitative and qualitative data collection, fluency in English and Swahili, and familiarity with the study sites. We have clarified that we conducted a prospective cohort study among AGYW aged 13–22 years, randomly selected in rural Gem and urban Nairobi informal settlements. (Line 31, page 2). We have also included in the Methods section, that AGYW were randomly sampled from a general population sampling frame (lines 144-148, page 7).

- Mention that “A total of 1081 AGYW…”, rather than starting a sentence with a number.

Thank you for the suggestion. We have corrected this in the abstract.(Line 44, page 2)

- Is self-efficacy in managing their health? This can be clarified.

Self-efficacy refers to AGYW’s belief in their ability to accomplish tasks, remain resilient in the face of adversity, and initiate coping behaviours when needed. It also relates to their confidence in managing their health and accessing necessary services. We measured general self-efficacy using a validated scale comprising ten questions that assess overall coping ability, problem-solving skills, and competence to meet goals. (Gourlay et al,2022). We have added the definition of self-efficacy in the methods section. (Line 250-254 Page 12)

- Address grammar throughout the abstract, including “This study compares…”.
Thank you for the suggestion. We have corrected this grammar throughout the abstract.

Introduction
- Need to clarify what “bear the brunt of the epidemic” means. Is it related to HIV infection incidence rates?

Yes, “bear the brunt of the epidemic” refers specifically to the significantly higher HIV incidence rates among AGYW compared to their male peers, as well as their disproportionate share of new HIV infections.

We have revised the paragraph to: In Eastern and Southern Africa, for example, AGYW on average experience three times the risk of HIV infection compared to their male peers, and are disproportionately affected by the epidemic in this region (Line 76-77, page 4)**.**

- For “inequalities and violence” did you mean to “mitigate/halt inequalities and violence”.

Thank you for the comment. We have revised the text to convey the intended meaning more clearly to: structural interventions to modify harmful gender norms, end gender-based discrimination, mitigate inequalities and violence (Line 85, Page 4)

- Briefly connect how DREAMS targets the UNAIDS pillar. Does it touch on all three components?

The UNAIDS HIV Prevention 2025 Road Map outlines five prevention pillars (HIV PREVENTION 2025 – ROAD MAP, UNAIDS 2022). DREAMS primarily targets the second pillar, HIV prevention among AGYW through delivery of a comprehensive package of biomedical, behavioral, and structural interventions. These include HIV testing and counselling, sexual and reproductive health services, gender-based violence prevention, and educational and economic support, all designed to address the multidimensional drivers of HIV risk in this population We have clarified this in the introduction section (Line 91-94, Page 5)

Methods
- Could you clarify the study design in the first sentence?

Thank you for the suggestion. We have split the paragraph into two, with subheadings Research Setting and Study Design. The first sentence in the study design section clarifies that our study design was a prospective observational closed cohort (Line 144, page 7).
- HIV testing services - how would it comprise HIV counselling services? Counselling should be grouped elsewhere.

HIV testing services in our study include both the diagnostic component (HIV testing) and the associated counselling, as these are routinely offered together as part of a service package. According to WHO guidelines, HIV testing services (HTS) comprise not only the provision of HIV tests but also pre-test information, post-test counselling, linkage to prevention, treatment and care services, and supportive services such as partner notification (WHO, 2015). National HIV programmes in Kenya have similarly integrated counselling into the HTS package to ensure individuals are supported before, during, and after testing (NASCO, 2015). In the context of the DREAMS initiative, HIV testing and counselling are intentionally delivered together as part of the biomedical intervention package (Saul et al, PLOS ONE, 2018). Given this established practice, we grouped HIV testing and counselling together in our analysis to reflect how they are implemented in practice within DREAMS and how they are delivered in the real-world. We have clarified in (Line 206, Page 10) that one of the primary interventions was HIV testing and counselling services.

- Please clarify how social support is defined as a binary variable. This appears to be an aggregate of 4 questions. Is a binary outcome more appropriate?

We defined social support as a binary variable generated using responses to four specific questions about female networks and access to safe social spaces to meet. A "high level of social support" was defined as a 'yes' response to at least three out of these four questions. Conversely, "lower levels of social support" were defined as 'yes' responses to between 0 and 2 questions. In the text where we define social support (lines xxx, page xxx), we have referred to the paper which gives more detail (reference 11) (Gourlay et al, BMJ Global Health, 2022)..

The four questions used to construct this measure were:

• "Is there a female in your community from whom you can borrow money in an emergency?"

• "Do you have at least one trusted female friend?"

• "Do you know a woman in your community, other than a mother or guardian, whom you could turn to if you had a serious problem?"

• "Do you have a safe and private place to meet with girls and young women who are like you?"

The decisions regarding this definition, including the cut-off(binary), were guided by descriptive analyses for each setting, which involved examining the distribution of 'yes' responses (overall, within age groups, and through cross-tabulation of all pairs of component questions) (Gourlay et al, BMJ Global Health , 2022)..

- A description of Self-efficacy can be more detailed. Is coping associated with mental health, social well-being, and academic/career challenges?

Thank you for the helpful comment. We have updated the description of self-efficacy in the paper (Line 250-254, Page 12). In brief, self-efficacy refers to an individual’s belief in their ability to manage tasks, cope with challenges, and achieve goals. It plays a key role in motivation, resilience, and problem-solving, and is relevant across multiple domains, including mental health, social well-being, education, and career development. Individuals with high self-efficacy are more likely to adopt coping strategies and remain resilient in the face of adversity. In this study, we used a validated ten-item scale to assess general self-efficacy, which reflects confidence in one’s ability to manage difficulties, problem-solving skills, and competence to meet goals (Gourlay et al,2022).

Results
- Aim to avoid starting sentences with numbers.

Thank you for the suggestion. We have corrected this in the results section.

- Table 2, regarding the p-value for Knowledge of HIV status. This should be for those who access the intervention? Rather than for those who were not invited?

Thank you for the correction. We agree that the p-value indicates whether there is evidence of a difference between those who accessed the intervention and those who were not invited to DREAMS. We have added a footnote that the p-values in Table 2 are based on the likelihood ratio test(LRT) and we have changed the placing of the p-value.

- Ensure that Supplementary tables have previously defined all acronyms used (I.e., HTS).

We have added footnotes in the supplementary tables to define all acronyms.(Line 532,page 30)

Discussion
- Based on the literature, are there reasons for increased condomless sex for sexually active individuals accessing the complete package?

No, based on literature there is no reason for this. The increase was observed only in Nairobi, and only among AGYW aged 15-17 years. Among AGYW aged 15-17 years in Nairobi, for those who were not invited to DREAMS the confidence interval was very wide for the percentage who had condomless sex in the previous 12 months (31% to 82%, point estimate 57.7%). This means that the comparison between invitees and non-invitees also has a very wide confidence interval. We have noted that the confidence interval is wide.

- What causes a discrepancy between urban and rural Kenya? Could a more detailed sociocultural context be provided to readers to help them better understand this? For example, what are the “fragmented social challenges” you’ve mentioned in urban areas?

We have provided context on the distinct characteristics between urban and rural Kenya. We have clarified in(Line 612-613, page 37) that rural Gem is characterised by strong community cohesion and more stable household structures, although the area is characterised by limited resources. In contrast, urban Nairobi is characterised by high population density, economic insecurity, household instability, and reduced community connectedness. AGYW in these settings are frequently engaged in informal work or caregiving responsibilities (Line 619-621 , page 38)

- Clarify “addressing the determinants of HIV risk among AGYW”. This is not clear - what are these determinants? These interventions are not necessarily targeted or tailored to specific risks, but rather constitute a series of broad-spectrum interventions

The phrase "addressing the determinants of HIV risk among AGYW" refers to determinants such as gender-based violence (GBV), limited access to secondary education, exclusion from economic opportunities, early marriage, multiple sexual partnerships, transactional sex, inconsistent condom use, and other experiences of violence and marginalization. We agree that DREAMS interventions are not necessarily targeted to specific individual risk factors. As outlined in the DREAMS Theory of Change, the programme uses a layered (combination of interventions) approach to deliver multiple interventions tailored to the needs of AGYW which is expected to act synergistically to address the broader behavioral, structural, and biomedical drivers of HIV risk. These interventions are designed to promote wider positive outcomes, including increased educational attainment, economic empowerment, and more equitable gender norms among AGYW, their families, and communities(Saul et al, PLOS ONE, 2018). We have changed the language from determinants to behavioural, biomedical and structural drivers of HIV risk(Line 600-601, page 36) to better reflect this broader focus.

- The "findings in context" section appears like a list. These findings could be better integrated throughout the "interpretation of findings" section instead.

We have integrated the two sections into one section as suggested.

- Please change “high levels” to “… high-level behavioural risk”.
Thank you for the suggestion. We have made the revision to high levels of behavioural risks (Line 623, page 38)

- Future steps should also be discussed. The manuscript highlighted the strength of partial interventions, but how will an optimal combination of the Partial DREAMS interventions be identified? Which individual intervention contributed most towards each outcome? This could be conducted in the future. Another consideration should be to offer a tailored set of DREAMS interventions for each person. Should tailored interventions be selected based on some form of risk-stratification model?

While our current analysis focused on evaluating the impact of DREAMS as a package aligned with how it is implemented in practice, we agree that future research could aim to understand which interventions contribute most significantly to specific outcomes. We have updated this in the discussion section (Line 737-740, Page 43)

Reviewer #2: The manuscript potentially makes a contribution to the HIV prevention literature by examining combination intervention packages rather than individual interventions. The focus on DREAMS is relevant considering the substantial financial investment into this initiative. Also, using data from established demographic surveillance systems provides a good base for the analysis.

The outcomes are generally well-defined and measured using appropriate timeframes.

Concerns requiring revision


1. Self-reported intervention receipt has been solely used without validation against program records or other objective measures. Self-reporting is subject to recall bias, bias due to perceived social desirability, or misunderstanding of the components of the intervention. Although acknowledged in the limitations, an assessment of concurrence between self-reported and program-records of intervention receipt would have been useful in avoiding any potential for misclassifying exposure status.

• If possible, validation of self-reported intervention receipt vis-à-vis program records for a subset of participants. It will also be important to acknowledge the limitations of self-reported exposure measurement and discuss the potential direction and magnitude of bias from miscalculation of exposure.

Thank you for the suggestion. Unfortunately, it was not possible to validate self-reported intervention receipt against program records for a subset of participants, as the DREAMS impact evaluation was conducted independently from the implementing DREAMS programme and we did not have access to individual-level service delivery records. However, DREAMS invitation was a formalised process, with clear eligibility criteria, and was therefore well understood by both implementers and participants. As such, we do not expect there to have been substantial misclassification of invitation status. We have clarified this in the limitation section (line 711-718, page 42).

.

2. The manuscript does not discuss missing data patterns, mechanisms, or handling strategies. There was no reporting of missing data percentages for key variables. Was missing data missing at random (MAR) or missing not at random (MNAR). What was the impact of missing data assumptions?

• Provide detailed missing data analysis including percentages missing for all key variables and assess patterns of missingness. Discuss potential impact of missing data on study conclusions.

There were no missing data for the outcomes and exposure in both sites with the exception of two confounding variables in Gem. For the two variables with missing data, we categorised missing responses as “unknown”, which reflected participants who could not be linked to the HDSS database from which the variables were derived. The proportion of missingness was relatively low for these two variables in Gem (education level, 21% & orphanhood, 24% as shown in existing tables(Table 1a)), and we did not conduct a complete case analysis due to concerns about generalizability (Allison, 2010). We have discussed that the proportion of missingness was low and unlikely to bias our findings (Line 726-730, page 43)

3. The exposure variable ‘invited by 2018 and accessed 0, 1, or 2 primary interventions’ suggests that accessing 0 intervention in equivalent to accessing 2. In the context of combination prevention, 2 interventions can be potentially significant in influencing outcomes depending on what and for whom.

• It would be helpful to reflect on how it may or not have impacted results and the question of the value of investing in two interventions if you can achieve same result with having only been invited to DREAMS.

We acknowledge that in the context of combination prevention, 2 interventions can be potentially significant in influencing outcomes. However, we categorized the "partial" package as accessing three interventions because this aligns with the UNAIDS definition of comprehensive combination prevention, which emphasizes the integration of biomedical, behavioural, and structural components. In contrast, those who accessed 0–2 interventions were less likely to have experienced this comprehensive layering. Our primary aim was to assess whether a “full package” was better than a “partial package” that was meaningful (that might be delivered as an alternative to a “full package”), and we considered that if a partial package were designed/implemented then it should still include 3 or more interventions (Line 233-236, Page 11). We acknowledge, however, that some AGYW within this group may have received an impactful subset of interventions.

4. There is insufficient detail on the timing of the intervention receipt relative to measurement of the outcome. Did all participants have the outcomes measured before completing interventions? Considering that the duration of intervention exposure varied across participants, there is a potential for reverse causation.

• Provide a timeline to show intervention implementation periods and outcome measurement windows. Analyses should be restricted to participants with adequate follow-up time after intervention completion. Discuss potential for reverse causation and strategies used to address it.

As stated in the Methods section, our primary exposure was a composite variable indicating whether an AGYW had been invited to participate in DREAMS interventions by 2018, and the number of primary interventions accessed by 2019. We have clarified that intervention uptake was assessed in the 2017/2018 and 2019 survey by asking AGYW whether they had accessed each intervention in the past 12 months (i.e., reflecting uptake before 2019).. All outcomes were measured using endline data collected in 2019 (Line 197-201, page 10). Given this design, all participants had their outcomes measured after the reported exposure period, making the risk of reverse causation minimal. We acknowledge that the duration of exposure to DREAMS interventions likely varied across individuals, which may influence the observed effects. We have provided additional clarification in the methods section

5. Numerous statistical tests have been conducted across multiple outcomes and subgroups without adjustment for multiple comparisons. Five primary outcomes tested in two settings with age stratification results in over 20 statistical tests. There may be an increased risk of Type I error (false positive findings)

• Apply appropriate multiple testing corrections, distinguish between primary and secondary analyses and acknowledge the increased risk of Type I error in the interpretation.

Thank you for raising this issue. The outcomes were all pre-specified as key secondary outcomes, and it is not usual to adjust for multiple comparisons in terms of assessing multiple outcomes. Adjustment for multiple comparisons is done when multiple exposures / explanatory variables are considered (for their effect on an outcome). In our evaluation, we had 3 comparison groups (non-invitees, DREAMS invitee and partial package, DREAMs invitee and full package), which is not many groups to compare. We also only did one a priori sub-group analysis, by younger or older age group.

We think it is not necessary to adjust for multiple comparisons, but we have taken care to be cautious in our interpretation of sub-group findings – especially when sub-group findings have wide confidence intervals.

6. The generalizability of findings beyond the study settings is not adequately discussed. Both settings are in Kenya.

• Expand discussion of to include factors that may influence generalizability. For example, comparison of the study settings to other implementation sites based on other characteristics beyond urban/rural. It would also be useful to reflect on the generalizability to other countries.

Thank you for this suggestion, which is important to address.

We have clarified in (Line 727-732 Page 43) that although our study was conducted in two Kenyan settings, rural Gem and urban informal settlements in Nairobi the findings are broadly generalizable to similar DREAMS implementation contexts in East Africa. These settings represent diverse profiles, with Gem reflecting a rural area with limited resources and Nairobi reflecting an urban informal settlement setting characterised by a high population density, poor infrastructure and environmental conditions as well as limited access to services.

Generalizability to other DREAMS countries may depend on the similarity of implementation, demographic risk profiles, and contextual factors such as education systems and health systems. Our results may not be generalisable to all DREAMS countries, but because they represent diverse implementation contexts the findings can contribute important insights for other settings in which DREAMS interventions are being implemented, and other complex interventions.

7. Strengthen connection between the findings and the DREAMS theory of change as well as more detailed discussion of implementation factors that may explain the differences.

Thank you for the suggestion. We have strengthened the link between our findings and the DREAMS theory of change. We have added details on this in the discussion section in (Line 691-696, Page 41) that our findings support DREAMS Theory of change, which highlight that a combination of biomedical, behavioral, and structural interventions can synergistically reduce AGYW’s HIV risk. We observed stronger effects among AGYW who received a more complete package of DREAMS interventions in both settings, supporting the idea that a combination of interventions is essential for shifting drivers of HIV risk.

8. More detailed discussion on the practical importance of observed effects, beyond statistical significance, is needed. Consider including discussion on the practical significance of observed effects for program implementation. Also, contextualizing the findings in relation to other evaluations of DREAMS.

Thank you for the suggestion. We have clarified in line .(Line 679-686, Page 41) that while some of the observed effects may appear modest, they are practically important. For example, increased knowledge of HIV status is a gateway to timely diagnosis, linkage to care, and prevention of HIV transmission. Improvements in social support and self-efficacy can enhance resilience, increase the uptake of services, and promote behaviour change. Similarly, a reduction in the number of sexual partners lowers the risk of HIV acquisition. These outcomes, though intermediate, are important for achieving long-term reductions in HIV incidence. These practical gains are essential for programme implementers aiming to optimize the real-world impact of DREAMS interventions in diverse settings.

9. Given the changing financial landscape, cost-effectiveness implications would be very useful.
We agree that assessing the cost-effectiveness of DREAMS would provide valuable insights, given the changing financial landscape and the need for sustainable programming. However, a cost-effectiveness analysis was beyond the scope of this paper, lessons can be drawn from other studies. For example, an analysis of the early roll-out of DREAMS in Kisumu, Western Kenya, found that delivery costs were higher in peri-urban areas than in an urban area, despite reaching similar numbers of AGYW, indicating potential differential cost implications by context (Okal et al., 2022).

Recommendation

The findings from this study have the potential to inform DREAMS programming and broader HIV prevention efforts for AGYW, but the limitations identified above should be addressed to ensure the conclusions are robust and reliable.

Reviewer #3: I commend the authors for a good job in presenting the DREAMS study analysis. My comments from the submission are as follows:
(1) What methods were used to prevent "contamination" of interventions to those who were not invited to participate in the study; or was this taken care of in the statistical analysis.

Thank you for the comment. The DREAMS implementing partners targeted and invited AGYW based on socio-economic and sexual risk vulnerability criteria such as those who were food insecure, out of school, or facing other socio-economic challenges (methods section, 184-190 , page 9) . While many AGYW in these settings face multiple vulnerabilities, only a subset of those meeting the eligibility criteria were invited to participate in DREAMS due to resource limitations.

Although the program aimed to restrict access to those formally invited, some AGYW who were not invited to DREAMS may have accessed certain interventions (including because they could be accessed without being a DREAMS invitee, e.g. HIV testing services, HIV and violence prevention education). We used a per-protocol analytical approach, classifying AGYW based on both their invitation to DREAMS and their actual receipt of DREAMS interventions. This approach allowed us to more accurately reflect real-world exposure and estimate the effect of the interventions as delivered. AGYW who were not invited to DREAMS received fewer interventions than DREAMS invitees (for example 39/224 (17%) in Gem and 84/436 (19%) in Nairobi received >=3 interventions; this is shown inS1a Table and S1b Table).

(2) The authors mentioned using DAG to draw causal assumptions between exposure and outcomes, the DAG was not presented in the paper. Can this DAG be presented in the paper?

We have now included the DAGs as one of the supplementary files ( Supplementary file S1 Figure)

(3) Authors used 2 methods for regression, multivariable regression and propensity score regression. Is there any particular reason for using these at the same time? If one method is better than the other, then it makes sense to use that method. If both are similar then presenting one method still makes sense?

We used multivariable logistic regression to estimate the association between DREAMS and each outcome, adjusting for measured covariates. This approach provides conditional estimates of effect using odds ratios to compare DREAMS invitees with non-invitees.

In contrast, the propensity score regression approach provides marginal estimates estimating the average causal effect of DREAMS on outcomes adjusted for the probability of exposure based on observed characteristics. This approach enables the comparison of counterfactual scenarios within a formal causal inference framework based on “potential outcomes”. This accounts for confounding due to non-random assignment of DREAMS (by balancing observed covariates, including confounding variables, between exposure groups) and mimics a randomised trial design.

We believe using both methods is helpful in our work. The multivariable logistic regression analysis approach shows associations while adjusting for covariates directly, and is familiar to readers, but its estimates are conditional and summarised as odds ratios (which can be difficult to interpret, when outcomes are not rare). The propensity score approach, on the other hand, offers marginal estimates that are more relevant for population-level inference, and enables comparisons to be made in terms of “risk differences” or “differences in the proportion with the outcome”, rather than using ratio measures for comparisons. (Line 285, Page 14 and 290-292, Page 14)

(4) When using propensity score regression for causal analysis there are several assumptions that are made. Can the authors state and show if these assumptions were met, and if not what methods were used to assess for the violation of assumptions. For example SUTVA (stable unit treatment value assumption) and exchangeability.

We have included supplementary material (Supplementary file S1 Text) and this is referred to in line 298-299 page 14, which describes the extent to which all causal assumptions including SUTVA (Stable Unit Treatment Value Assumption) and exchangeability are likely to hold in our study context.

(5) Tables can be improved so that the results presented are better to read and understand.

We have ensured that the results are presented better.

Reviewer #4: The manuscript is well constructed, cohesive and delineates the principal interventions targeting AGYW.

Please provide additional details on the following:


1) Please clarify the mean (or median) age difference between questionnaire administrators and adolescent girls and young women (AGYW) who provided informed consent.
The median age difference between question administrators was 6 years in Gem and 4 years in Nairobi.

2) Describe the physical setting in which the questionnaires were administered e.g., a private room versus a communal household space, and indicate whether parents, caregivers, or other AGYW were within visual or auditory range during enrolment and data collection.

All interviews were conducted in-person, primarily within the AGYW’s household. To ensure privacy and confidentiality, interviews were held in a private space within the home, away from other household members whenever possible. Field interviewers were trained to identify and request the most appropriate and private location for the interview such as a separate room, outdoor space depending on the structure of the household. Efforts were made to ensure that parents, caregivers, or other AGYW were not within visual or auditory range during enrolment and data collection. We have added more details on this in the data collection in the methods sections (Line 157-158, page 8)

3) Detail the procedures used to safeguard confidentiality when sensitive items were addressed.

We implemented several procedures to ensure confidentiality and protect participants' privacy, particularly when addressing sensitive topics.

Trained and experienced interviewers: All participants were interviewed by trained data collectors who were fluent in the local language, familiar with the cultural context, and experienced in handling sensitive topics. Interviewers were trained on ethical research practices, including maintaining confidentiality and creating a non-judgmental and safe environment for AGYW. Before each interview, participants were informed about the purpose of the study, their right to decline or skip any question, and the strict confidentiality of their responses.

Anonymized data collection: No personally identifiable information (e.g., names, addresses, phone numbers) was collected in the main dataset used for analysis. Each participant was assigned a unique study ID, and all analyses were conducted using anonymized datasets.

Data were collected using secure electronic tablets with password protection and encrypted data storage. The system offered enhanced security compared to paper-based methods, as it reduces the risk of physical data loss or unauthorized access. Data were synchronized daily to a secure server with restricted access.

Access to the data was limited to authorized study personnel only. Data were stored on password-protected and encrypted servers, and all data handling procedures adhered to institutional and national data protection guidelines. We have provided a supplementary text that describes the study procedures in detail (Supplementary S2 Text).

4) Outline the methods employed to verify response accuracy and specify any quality-control metrics applied.

To ensure the accuracy of AGYW responses, we implemented multiple quality assurance procedures during data collection. All data collectors underwent intensive training on the study protocol, questionnaire content, ethical conduct, and appropriate probing techniques. Mock interviews and field practice sessions were conducted during the training to ensure consistency and correct interpretation of questions.

We used a tablet-based electronic data capture system programmed with built-in skip patterns, logic checks, and range validations to prevent illogical or inconsistent responses. These automated checks minimized data entry errors at the point of collection.
Data collected each day were synchronized to a secure central server and study team conducted quality checks. These included checking for missing data, inconsistencies, and outlier values. Any flagged records were reviewed and, when needed, followed up with the interviewer. Field supervisors regularly conducted spot checks and random checks to confirm that interviews had been conducted as reported, and that protocols were being followed.
Regular debriefing meetings were held with field staff to discuss challenges, clarify questionnaire items, and share feedback from data quality reviews. Interviewers received refresher guidance or corrective instructions as needed. We have provided a supplementary text that describes the study procedures in detail (Supplementary S2 Text).

5) Explain the analytical or design strategies adopted to adjust for confounding variables such as participant age, mental-health status, cultural practices, and religious affiliation during risk profiling and stratification.

A census-based approach known as the Girl Roster was used to map AGYW across the study areas, to guide which AGYW would be invited to participate in DREAMS (Chimbindi et al., *PLOS ONE*, 2018; Population Council, 2016). This tool collects key information on age, marital status, childbearing, schooling, and household arrangements, enabling stratification of AGYW into risk groups. Based on these profiles and supplemented by local insights from implementing partners AGYW deemed vulnerable (e.g., out of school, married young, or with children) were invited to DREAMS. This involved door-to-door recruitment followed by structured enrolment interviews.(Line 184-190, page 9)

To address potential confounding in the impact evaluation analysis using our study cohort data, we constructed a propensity score model that estimated AGYW probability of being invited to DREAMS. The propensity score was derived using a logistic regression model that included a set of covariates informed by Directed Acyclic Graphs (DAGs). These DAGs were developed based on prior knowledge of how DREAMS was implemented and targeted, specifically, the individual and household-level characteristics that determined both invitation to DREAMS and the outcomes of interest (e.g., age, schooling, marital status, socio-economic status, food insecurity).By incorporating these covariates into the propensity score model, we aimed to balance the exposure groups (not invited to DREAMS, invited and received “partial package” and invited and received “complete” package) on observed confounders, thereby reducing confounding bias. This allowed us to estimate the causal effect of DREAMS on key outcomes, under the assumption of no unmeasured confounding. We have added information on the analysis in the methods section(Line 297-298,Line 304-307 page 14)

6) Given the broad eligibility age range of 10–24 years, how were questions related to lifetime number of sexual partners structured to ensure age-appropriateness and cognitive suitability for younger participants? Please describe any adaptations made to account for developmental differences across early, middle, and late adolescence.

Although the broader DREAMS programme targets AGYW aged 10–24 years, this analysis focused on a sub-sample of AGYW aged 13–22 years. Questions about sexual behavior, including lifetime number of sexual partners, were only asked if the AGYW indicated that they had ever had sex. This minimised unnecessary or inappropriate questioning among younger participants who had not initiated sexual activity. Sensitive items were phrased using appropriate and non-judgmental language. Interviewers received training on how to approach sensitive topics with adolescents of different ages, including strategies for building rapport, ensuring privacy, and adjusting tone and pace based on the age of the AGYW. Interviews were conducted in private settings to help participants feel comfortable and safe when responding to sensitive questions. By implementing these adaptations, we aimed to create an appropriate environment for AGYW while maintaining the integrity of data collection. We have provided a supplementary text that describes the study procedures in detail (Supplementary S2 Text).

Reviewer #5: Overall comments
The authors provide a comprehensive evaluation of a combination of interventions that can optimize HIV prevention among adolescent girls and young women (AGYW) in Kenya. This work contributed to the evidence base on HIV programming in resource-limited settings. However, this manuscript could be made stronger and could benefit from streamlining of the content and improved clarity.

See comments below for consideration
Introduction
Clearly outlined with excellent contextual information. However, it’s a bit long and would benefit from a reduction in some content that can be moved to the methods section. For example, the DREAMS selection process and package lists could be moved to the methods section.

We have moved the paragraph on the DREAMS intervention packages and DREAMS selection process to the methods section with the subheading DREAMS implementation context and DREAMS interventions (Line 161, page 8)

Methods
Well-structured design applied across two diverse settings. Detailed explanation and account of the methods provided. While the use of statistical techniques is appropriate and required, the analysis section is overly lengthy and somewhat technical, which may limit some audiences. Perhaps consider summarizing the key analytic steps more concisely to improve clarity and reader engagement.

We appreciate this helpful suggestion. While it is important for us to retain the full analytic detail, we have revised the section to improve the flow and readability. Specifically, we have broken the description into shorter paragraphs so that the key analytic steps are more clearly highlighted. This structure allows us to maintain the necessary level of detail while also making the text easier to follow for readers

Ethical considerations-where were the consent forms stored?

Signed consent forms were securely stored in locked filing cabinets at the study site offices with access limited to authorized study personnel. These forms were not linked to the analytical dataset, which contained only anonymized data, to ensure participant confidentiality. We have provided a supplementary text that describes the study procedures in detail (Supplementary S2 Text).

Discussion
The findings are appropriately synthesized and interpreted within the broader literature.
Line 517-555 provides a summary of the findings, though long. Please revise to be more concise and focus on the key findings.

Thank you for the feedback. We have revised this section and reduced and focused on the key findings.(Line 552-581 , page 35 &page 36)

Line 551-552- Why do you think the partial package was effective than the complete package in Nairobi?

Thank you for the comment. To clarify, we found that receiving a partial package of DREAMS interventions (3–4 interventions in Nairobi) appeared to be less effective than receiving the complete package (5 interventions) in Nairobi, though in some outcomes, its effectiveness was similar to the complete package observed in rural Gem. This is highlighted in the conclusion section of the abstract (Line 55-57 page 2 and line 592-594 page 37 and 598-600 page 37)

Line 586-593 Briefly discuss how the intervention tailoring by age can influence these patterns

We have updated this in the discussion section of the paper in (Line 649-652 page 39) and discussed how intervention tailoring by age may have influenced the observed patterns across outcomes. DREAMS intervention delivery was stratified by age to align with the developmental stage and risk profiles of AGYW at different points in adolescence and young adulthood.

In brief, DREAMS applied a layering approach delivering multiple interventions and the specific combination of services to be layered was explicitly defined by age categories: 10–14, 15–19, and 20–24 years. A minimum or "primary package" of DREAMS services was outlined for each age group to ensure that core interventions were aligned with the needs of that cohort. These primary interventions were expected to be received by all AGYW in a given age group, while additional "secondary" interventions were tailored based on individual circumstances and vulnerabilities(Chimbindi et al, PLOS ONE, 2018).These age-based differences in intervention delivery likely contributed to the difference in impacts observed across outcomes by age groups.

Line 594-596: Provide details on how the sustainability of the effects in the long term may rely on family, community, and institutional support. Integration?

We have provided additional detail in (Line 664-670 page 40) that the sustainability of the girl-centric approach may depend on broader system-level integration such as the involvement of family, community, and institutional support. These structures can reinforce and sustain the benefits of DREAMS interventions beyond the individual level.

6. PLOS authors have the option to publish the peer review history of their article ([what does this mean?](https://track.editorialmanager.com/CL0/https:%2F%2Fjournals.plos.org%2Fglobalpublichealth%2Fs%2Feditorial-and-peer-review-process%23loc-peer-review-history/1/010f0197effff3b2-6e2a1771-a554-472c-8e6e-ae02c22b511b-000000/arVEAlTBf8ppiIOn5GQ4bF7ZfKw123rhapTyuUiExZ0=218)). If published, this will include your full peer review and any attached files.
Yes

**Do you want your identity to be public for this peer review?** If you choose “no”, your identity will remain anonymous but your review may still be made public.


For information about this choice, including consent withdrawal, please see our [Privacy Policy](https://track.editorialmanager.com/CL0/https:%2F%2Fwww.plos.org%2Fprivacy-policy/1/010f0197effff3b2-6e2a1771-a554-472c-8e6e-ae02c22b511b-000000/zMw7jInvVH_ygr-WyIWgVWYgqCrFjItNjofIwlLtfng=218).

Reviewer #1: No

Reviewer #2: No

Reviewer #3: No

Reviewer #4: No

Reviewer #5: No
